# A Mechanism-based QSTR Model for Acute to Chronic Toxicity Extrapolation: A Case Study of Antibiotics on Luminous Bacteria

**DOI:** 10.1038/s41598-017-06384-9

**Published:** 2017-07-20

**Authors:** Dali Wang, Yue Gu, Min Zheng, Wei Zhang, Zhifen Lin, Ying Liu

**Affiliations:** 1 State Key Laboratory of Pollution Control and Resource Reuse, College of Environmental Science and Engineering, Tongji University, Shanghai, 200092 China; 2 Post-doctoral Research Station, College of Civil Engineering, Tongji University, Shanghai, 200092 China; 3 College of Fisheries and Life Science, Shanghai Ocean University, Shanghai, 201306 China; 4 School of Resource and Environmental Engineering, East China University of Science and Technology, Shanghai, 200237 China; 5 Collaborative Innovation Center for Regional Environmental Quality, Beijing, China; 6 Shanghai Key Laboratory of Chemical Assessment and Sustainability, Shanghai, China

## Abstract

The determination of the chronic toxicity is time-consumed and costly, so it’s of great interest to predict the chronic toxicity based on acute data. Current methods include the acute to chronic ratios (ACRs) and the QSTR models, both of which have some usage limitations. In this paper, the acute and chronic mixture toxicity of three types of antibiotics, namely sulfonamides, sulfonamide potentiators and tetracyclines, were determined by a bioluminescence inhibition test. A novel QSTR model was developed for predicting the chronic mixture toxicity using the acute data and docking-based descriptors. This model revealed a complex relationship between the acute and chronic toxicity, i.e. a linear correlation between the acute and chronic lg(−lgEC50)s, rather than the simple EC_50_s or −lgEC_50_s. In particular, the interaction energies (E_bind_) of the chemicals with luciferase and LitR in the bacterial quorum sensing systems were introduced to represent their acute and chronic actions, respectively, regardless of their defined toxic mechanisms. Therefore, the present QSTR model can apply to the chemicals with distinct toxic mechanisms, as well as those with undefined mechanism. This study provides a novel idea for the acute to chronic toxicity extrapolation, which may benefit the environmental risk assessment on the pollutants.

## Introduction

The chronic toxicity can better reflect the environmental risks of pollutants than the acute toxicity, since the organisms in the real environment are commonly subjected to long-term exposure of pollutants. However, it remains challenging to obtain the chronic toxicity directly through experimental methods, comparing to a flood of acute toxicity data that have been collected. This is because the chronic tests are usually characterized by long test cycle, complicated operation and high cost^1^. An effective solution to these problems is to extrapolate the chronic toxicity from the acute data that are easily obtained experimentally^2, 3^. Therefore, the underlying difference and connection between the chronic and acute toxicity should be investigated, which may provide easy and quick methods for acute to chronic toxicity extrapolation, and help establish chronic toxicity database for the environmental risk assessment.

At present, the acute to chronic toxicity ratios (ACRs) are commonly used for acute to chronic toxicity extrapolation. The ACR of a chemical is obtained as the ratio of the median lethal concentration (LC_50_) and the chronic maximum acceptable toxicant concentration (MATC)^1–3^. It is assumed that the ACR of a certain chemical is a constant for different species, which is calculated using the observed acute and chronic toxicity data with one species and then used to predict the chronic toxicity for another that is exposed to the same chemical^4^. This method is easy to perform and has been used in deriving US National Ambient Water Quality Criteria (NAWQC)^5^ as well as in risk assessments in many countries^6^. However, this method is problematic, for the ACR of a chemical may vary with many factors, such as the chemical modes of action (MOA), the model organisms and the experimental conditions^3^. Only when the MOA of a chemical is narcotic, can its ACR be a constant^7, 8^. Otherwise, the ACRs of the chemicals (i.e. the reactive compounds) can vary in a wide range (up to four orders of magnitude difference)^2, 3^, which makes it difficult to choose an appropriate ACR value for the chronic toxicity extrapolation and sometimes results in a under- or over-estimation of the chronic toxicity.

Quantitative structure-toxicity relationships (QSTR) severs as an important tool for the chronic toxicity prediction in risk assessment^9^. To date, a great many QSTR models have been reported for the chronic toxicity prediction, covering a variety of organisms and chemicals^10–13^. And various chemical properties have been introduced as the molecular descriptors for the QSTR models, such as logK_ow_^8, 10^, E_LUMO_^14, 15^ and E_HOMO_^16, 17^. In particular, the acute toxicity of chemicals was involved as one of the molecular descriptors in some QSTR models. For instance, Jiang *et al*.^18^ developed a chronic QSTR model that linked the 24 h toxicity [lg(1/EC_50–24 h_)] of antibiotics on luminescent bacteria to the 30 min toxicity [lg(1/EC_50–30 min_)] and other five descriptors. Likewise, in the report of Zou *et al*.^19^, the lg(1/EC_50–24 h_) of antibiotics on the luminescent bacteria can also be related to the lg(1/EC_50–30 min_). However, the QSTR model predictions also have some limitations, for instance, they can only apply to the chemicals with the similar structures or the same action mechanisms^20^. Moreover, few of the researches consider the chronic effects at the population level from the chemical ecology perspective, e.g. the potential effects of the chemicals on the bacterial quorum sensing (QS) systems.

QS is a cell-cell communication by which bacteria coordinate the expression of certain genes using small signal molecules (autoinducers, AIs)^21^. During their normal physiological process, the bacteria secrete AIs into the surrounding environment, which accumulate to a threshold concentration and then re-enter the bacterial cells, regulating gene expression and a series of bacterial behaviors, such as the biofilm formation^22^ and luminescence^23^. The acute toxicity test with the luminescent bacteria usually takes only 15 to 30 min, while the chronic toxicity test takes 24 hours or even longer. Therefore, during the acute test, the action of the chemicals on the bacterial QS systems can be neglected, whereas during the chronic test, the chemicals may exert considerable effects on the bacterial QS systems and thereby the bacterial behaviors. So we assume that the acute toxicity on the bacteria is caused by the interference with the structures and functions of the biomolecules, while the chronic toxicity should include the effects on the bacterial QS communications. This assumption provides a possibility to extrapolate the chronic toxicity data from the acute ones by using parameters that are concerning the bacterial QS systems. This is what we are going to explore in the present paper.

In this study, the acute (15 min) and chronic (24 h) toxicity of fifteen antibiotics, inclusive of sulfonamides (SAs), sulfonamide potentiators (SAPs) and tetracyclines (TCs), were determined by a bioluminescence inhibition test based on *Vibrio fischeri* (*V*. *fischeri*), both individually and in combination. The differences between the mechanisms of the acute and chronic toxicity were explained from the perspective of bacterial QS communication. A QSTR prediction model was then constructed for the chronic toxicity, by using the acute toxicity and the molecular docking-based descriptors. The current study provides a novel method in predicting the chronic toxicity of antibiotics, which may help make environmental risk assessment on the pollutants.

## Results

### Individual toxicity of the antibiotics

#### Comparison between the acute and chronic toxicity of individual antibiotics

The acute and chronic toxicity (−lgEC_50_) of the individual antibiotics were listed in Table 1. In general, the chronic −lgEC_50_s were greater than the acute ones, indicating a greater action of the antibiotics on the bacteria during the chronic test. Moreover, the differences between the acute and chronic −lgEC_50_s varied with the chemical type. The largest differences between the acute and chronic −lgEC_50_s were observed with the two SAPs, i.e. OMP and TMP. Their acute −lgEC_50_s were 3.29 and 3.22, while the chronic −lgEC_50_s were 6.51 and 6.48. The differences between the acute and chronic −lgEC_50_s for them were 3.12 and 3.26, respectively. With respect to SAs, the differences between the acute and chronic −lgEC_50_s were moderate, ranging from 0.41 (SDX) to 1.81 (SMP); whereas for TCs, only slight difference were observed between the acute and chronic −lgEC_50_s, ranging from 0.11 (MH) to 0.71 (CH).

**Table 1:** Information on the test chemicals.

Chemicals	Abbr.	${\boldsymbol{-}}\text{lgE}{{\bf{C}}}_{{\bf{50}}}^{{\bf{a}}}$ (mol/L)	K_a_	${\boldsymbol{-}}\text{lgE}{{\bf{C}}}_{{\bf{50}}}^{{\bf{c}}}$ (mol/L)	K_c_	Ebind^a^	Ebind^c^ (kcal/mol)	
						Luc	Targets*	LitR
Sulfadiazine	SD	3.03	44.9	4.22	252.57	−31.80	−26.58	−25.46
Sulfadoxine	SDX	3.64	65.71	4.05	336.69	−33.77	−29.84	−31.60
Sulfisoxazole	SIX	3.54	61.5	4.59	312.87	−34.18	−34.41	−26.31
Sulfameter	SM	2.87	57.03	4.30	278.21	−39.32	−24.81	−30.59
Sulfamonomethoxine	SMM	3.18	61.17	4.86	299.76	−34.36	−26.23	−27.75
Sulfamethoxypyridazine	SMP	2.99	51.97	4.80	248.43	−34.81	−28.80	−29.96
Sulfamethoxazole	SMX	3.61	71.35	5.03	350.84	−27.62	−29.75	−25.85
Sulfamethazine	SMZ	2.77	37.74	4.30	243.47	−33.85	−30.91	−30.85
Ormethoprim	OMP	3.39	176.07	6.51	1077.8	−37.89	−35.81	−28.94
Trimethoprim	TMP	3.22	169.51	6.48	1006.1	−38.28	−38.23	−31.79
Chlortetracycline hydrochloride	CH	4.22	124.11	4.93	1155.3	−59.22	−36.31	−41.69
Doxycycline hyclate	DH	4.45	155.31	4.80	1099.6	−51.62	−40.38	−38.53
Minocycline chloride	MH	4.31	89.23	4.42	834.02	−60.55	−32.45	−40.03
Oxytetracycline hydrochloride	OH	3.76	113.73	3.94	1063.1	−50.37	−40.27	−40.82
Tetracycline hydrochloride	TH	4.06	121.98	4.30	1155.3	−51.84	−33.68	−39.43

^a^The target proteins for SAs, SAPs and TETs were DHPS, DHFR and 30 s subunit of ribosomes, respectively.

**Figure 1:**
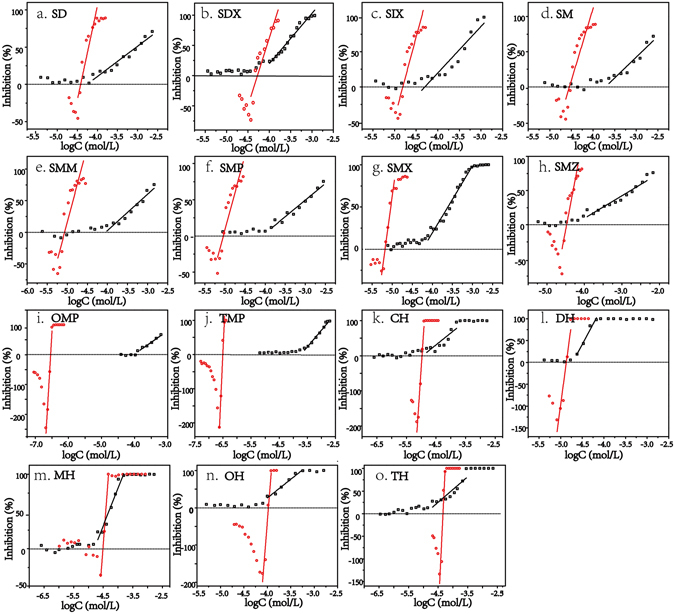
Dose-effect curves for the acute (black) and chronic (red) toxicity of the individual antibiotics.

**Figure 2:**
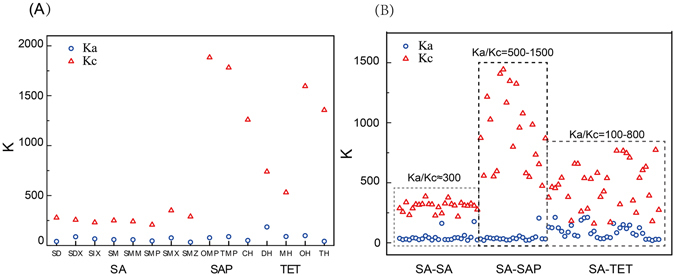
Comparisons between Ka and Kc of the single chemicals (**A**) and the binary mixtures (**B**).

Figure 1 shows the comparisons of the dose-response curves for the acute and chronic toxicity. In particular, the antibiotics at low concentrations presented stimulatory effects on the bioluminescence in chronic test, which manifested hormetic characteristics^24^. Besides, the slope for the chronic toxicity (K_c_) was significantly greater than that for the acute toxicity (K_a_), which suggested that the effects of the antibiotics in the chronic test varied from no observed effect to complete (100%) inhibition within a narrow concentration range. For example, in the chronic test, the effects of TMP (Fig. 1j) on *V*. *fischeri* varied from no observed effect to complete inhibition with the concentration increasing by only 1.30 × 10^−7^ mol/L; while in the acute test, the concentration increase for TMP was almost two orders of magnitude, with the log concentration ranging from −4.5 to −2.5 (Fig. 1j). Although K_c_ was greater than K_a_ for all of the chemicals, their absolute values varied vastly with the chemical type (see Fig. 2A). For SAs, K_a_ values distributed within 37.71–71.35, while K_c_ values ranged from 243.47 to 350.84. With respect to SAPs and TCs, the K_a_ values fell in the range of 100–200, whereas the K_c_ values were greater than 1000, with only one exception MH (the K_a_ and K_c_ were 89.23 and 834.02, respectively).

The differences between the acute and chronic −lgEC_50_s, as well as K_a_ and K_c_ reflected varying responses of *V*. *fischeri* to the antibiotics in the acute and chronic test, implying a much greater susceptibility to the chronic exposure than to the acute exposure. At least two reasons may account for these differences, one is the exposure time, and the other is the varying toxic mechanisms in the acute and chronic actions.

#### Mechanisms for the acute and chronic toxicity of individual antibiotics

**Figure 3:**
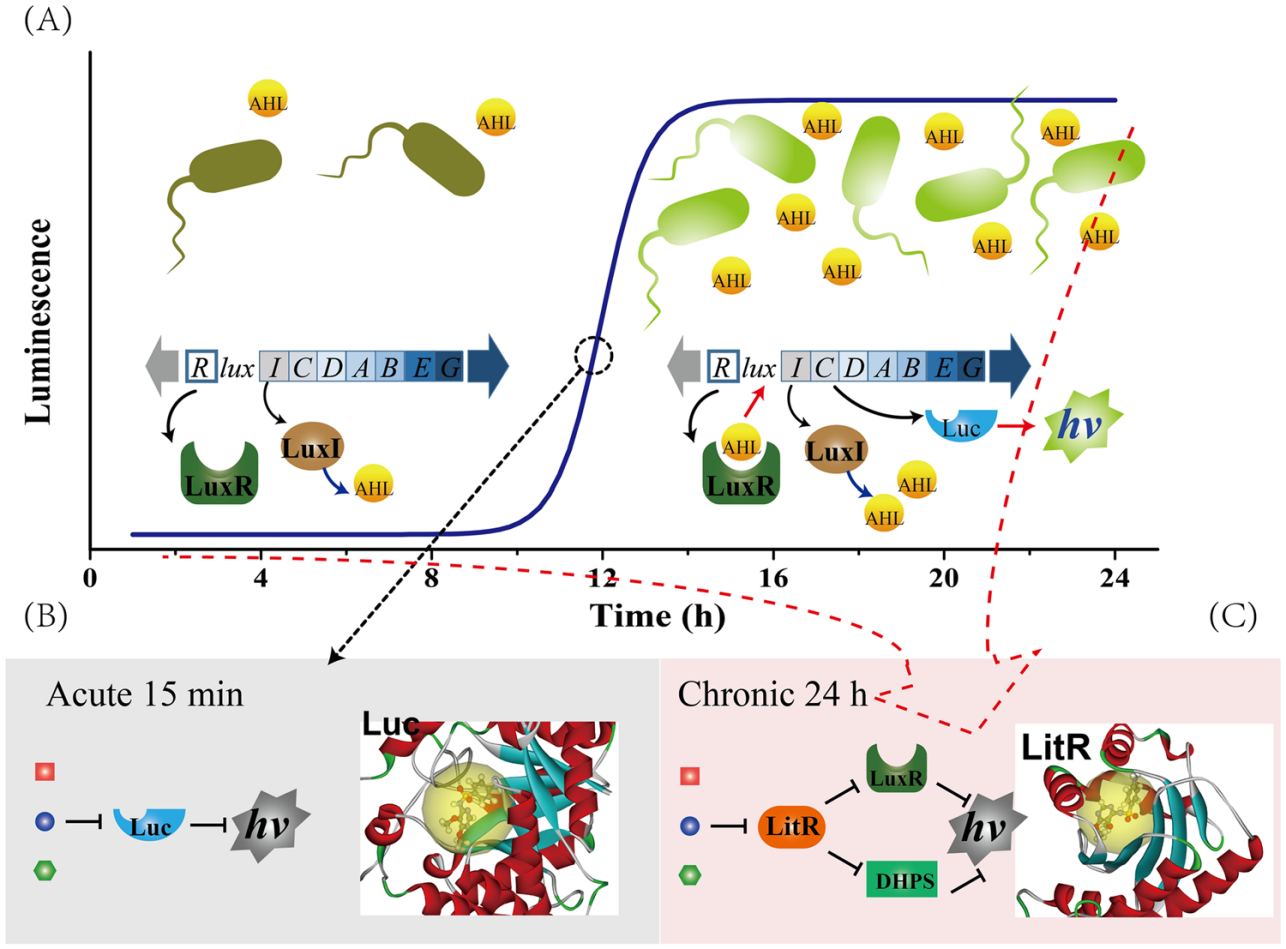
Mechanisms for the acute and chronic toxicity of individual chemicals. (**A**) The luminescence curves of *V*. *fischeri* during 0–24 h. From 0–10 h, there was no QS communication among the bacteria, since the bacteria and the AIs were at low concentrations. After 10 h, the AIs around the bacteria achieved the threshold concentration and triggered on the bacterial QS communication. (**B**) In the acute test, the antibiotics primarily target the luciferase (Luc) to inhibit the bioluminescence. (**C**) In the chronic test, the antibiotics acted on LitR to inhibit the QS communication and thereby the bioluminescence.

The acute test in the current research takes only 15 min, therefore the acute toxicity of the antibiotics was primarily due to their interference with the light emitting process, and the luciferase might act as the main target of antibiotics^25^, as shown in Fig. 3B. While the chronic test takes 24 h, during which the bacteria grow from extremely low density to high density (stationary phase), as shown in Figure S1 in the supporting information. During this period, the antibiotics could on the one hand bind with their target proteins^26^ (DHPS, DHFR and 16S rRNA, respectively), killing or inhibiting the bacterial growth, on the other hand affect the bacterial QS communications (see Figure S2 for detailed information on QS of *V*. *fischeri*).

**Figure 4:**
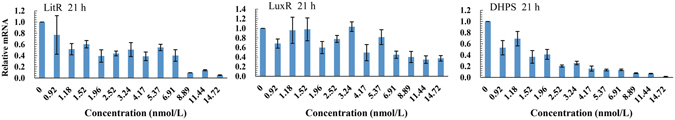
Expression of related proteins in the QS systems of *V*. *fischeri* upon exposure to SCP.

The expression of the QS-related genes was determined upon exposure to SCP in the chronic toxicity, in order to investigate its influence on the bacterial QS communication. As depicted in Fig. 4, SCP presented concentration-dependent inhibition on the expression of *litR*, *luxR*, and *dhps*. This suggested that the bacterial QS communication were considerably influenced by the exogenous drugs. The decrease in the *luxR* mRNA expression was probably induced by the depression on the *litR* expression, since the *litR* locates in the upstream of *lux* genes. In light of this, the actions on the LitR proteins by the antibiotics were actually prior to their actions on the LuxR (Fig. 3C). Therefore, the influences of the antibiotics on the LuxR proteins can be represented by the influences on the LitR. In addition, the expression of *dhps* mRNA was also inhibited by SCP in a similar concentration-dependent mode (Fig. 4). The same change trend of *litR* and *dhps* with the SCP concentration indicated that LitR probably participates in regulating the *dhps* expression. Therefore, the influences of the antibiotics on their target proteins can also be related to their actions on LitR (Fig. 3C).

#### Quantitative relation between K_a_ and K_c_ for individual antibiotics

Based on the above analysis, the antibiotics primarily target luciferase in acute actions, while may influence both their target proteins and the bacterial QS systems in chronic actions. Therefore, the higher sensitivity of *V*. *fischeri* in the chronic test was probably due to the interference of antibiotics with the bacterial QS communication, and the differences between K_a_ and K_c_ can be explained by the interaction between the antibiotics and the QS-related proteins.

Herein, we introduced lgK_c_/K_a_ to represent the difference between K_c_ and K_a_. This parameter may reflect the difference of the *V*. *fischeri* sensitivity to the antibiotics in acute and chronic test. A greater value of lgK_c_/K_a_ suggested a larger difference in the sensitivities between acute and chronic test. It was then found that lgK_c_/K_a_ for the individual antibiotics showed a good correlation (R^2^ = 0.827) with E^Luc^ and E^LitR^ (Equation 1) through the multiple linear regression analysis.

1 $${\rm{lg}}\,\frac{{K}_{c}}{{K}_{a}}=0.275-0.004{{\rm{E}}}^{{\rm{Luc}}}-0.011{{\rm{E}}}^{{\rm{LitR}}}$$

n = 15, R^2^ = 0.827, RMSE = 0.053, F = 28.696, P = 0.000, ${{\rm{Q}}}_{{\rm{loo}}}^{2}$ = 0.748, RMSE_loo_ = 0.057, ${{\rm{Q}}}_{{\rm{lto}}}^{2}$ = 0.742, RMSE_lto_ = 0.058.

${{\rm{Q}}}_{{\rm{loo}}}^{2}$ and ${{\rm{Q}}}_{{\rm{lto}}}^{2}$ are the cross-validated squared correlation coefficients from leave-one-out (LOO) and leave-two-out (LTO) cross-validation, respectively. The high ${{\rm{Q}}}_{{\rm{loo}}}^{2}$ = 0.748 and ${{\rm{Q}}}_{{\rm{lto}}}^{2}$ = 0.742 suggested a good internal validation. (The external validation was not performed for the individual toxicity data due to the limited size of the data).

Besides, it was found that the lgK_c_/K_a_ also had a good correlation with E^Luc^ and E^T^ (Ebind with the respective targets). As indicated by Equation 2, the determination coefficient (R^2^) of this model was 0.819, which was slightly lower than that of Equation 1.

2 $${\rm{lg}}\,\frac{{{\rm{K}}}_{{\rm{c}}}}{{{\rm{K}}}_{{\rm{a}}}}=0.300-0.009{{\rm{E}}}^{{\rm{Luc}}}-0.004{{\rm{E}}}^{{\rm{T}}}$$

n = 15, R^2^ = 0.819, RMSE = 0.054, F = 27.150, P = 0.000, ${{\rm{Q}}}_{{\rm{loo}}}^{2}$ = 0.725, RMSE_loo_ = 0.059, ${{\rm{Q}}}_{{\rm{lto}}}^{2}$ = 0.718, RMSE_lto_ = 0.060.

It should be noticed that E^T^ in Equation 2 was obtained with distinct proteins for different drugs, namely DHPS for SAs, DHFR for SAPs and 30 s subunit of ribosomes for TCs, respectively. Therefore, the E^T^ values varied with not only the composition of the chemicals but also the target proteins. For this reason, the E^T^ in Equation 2 will not be able to discriminate the toxic effect of a defined drug when the target protein varied. In this sense, the moving average approach^27–30^ was employed to generate new descriptors ΔE_*ij*_, as follows:

3 $${{\rm{\Delta }}{\rm{E}}}_{ij}={{\rm{E}}}_{i}-{({{\rm{E}}}_{ij})}_{avg}$$

In this equation, E_*i*_ denotes the interaction energy of drug *i* with the protein, j denotes the target (e.g., DHPS and DHF), and (E_*ij*_)_*avg*_ is the average value of Ebind for all drugs with the same j. Using this new descriptor, another QSAR model for lgK_c_/K_a_ was obtained with high R^2^ (0.803) as below:

4 $${\rm{lg}}\,\frac{{{\rm{K}}}_{{\rm{c}}}}{{{\rm{K}}}_{{\rm{a}}}}=0.802-0.010{{\rm{\Delta }}{\rm{E}}}^{{\rm{Luc}}}-0.003{{\rm{\Delta }}{\rm{E}}}^{{\rm{T}}}$$

n = 15, R^2^ = 0.803, RMSE = 0.056, F = 24.490, P = 0.000, ${{\rm{Q}}}_{{\rm{loo}}}^{2}$ = 0.715, RMSE_loo_ = 0.060, ${{\rm{Q}}}_{{\rm{lto}}}^{2}$ = 0.709, RMSE_lto_ = 0.061.

In Equations 1, 2 and 4, E^Luc^ represents the effects of antibiotics on luciferase in acute test, while E^LitR^ and E^T^ reflect the effects of antibiotics in chronic actions. The good relationships between them and lgK_c_/K_a_ suggested that the distinct targets in the acute and chronic test may account for the differences between K_a_ and K_c_. Furthermore, as noted above, LitR may involve in regulating *dhps* genes, so the bind of the antibiotics with LitR may affect their actions on the target proteins (Fig. 3C). Consequently, the interaction between the antibiotics and the target proteins (*i*.*e*., E^T^) can be represented by E^LitR^.

In Equation 1, it was seen that the lgK_c_/K_a_ values were negatively correlated to E^LitR^. This is likely because the lower Ebind values represented stronger interaction between the drugs and the proteins, which resulted in larger differences between the acute and chronic actions. Based on the above analysis, it can be deduced that the difference between K_c_ and K_a_ for individual antibiotics was due to the distinct targets in acute and chronic test, and their difference (represented by lgK_c_/K_a_) can be quantitatively characterized by their interaction energy with the proteins (Ebind).

### Combined toxicity of the binary antibiotic mixtures

#### Different joint effects of the antibiotic mixtures

The combined toxicity of the binary mixtures of SA-SA, SA-SAP and SA-TC were determined in both acute and chronic test, the TU of the mixtures were listed in Table S1 in Supporting Information. According to Table S1, all of the mixtures presented antagonistic joint effects in the acute toxicity test, with TU values greater than 1.2. The results were consistent with the findings of Zou *et al*.^19^, in which SAs and TMP showed antagonistic joint effects on the bioluminescence of *Photobacterium phosphoreum* (15 min) with TU ranging from 1.44 to 4.54. This was because the components in the mixtures both target the luciferase in the acute actions (as depicted in Figure S3), and the competition between them may hamper their interaction with the proteins and thus reduced their inhibition on the bioluminescence. In the chronic test, the joint effects of the antibiotic mixtures varied with the type of the components, exhibiting either synergism or addition. For the mixtures of SA-SA, TU values varied from 0.8 to 1.19, suggesting simply additive effects between the two components. This is similar to the results in Fang *et al*.^31^ that the binary mixtures of SAs presented additive effects on *E*. *coli* (12 h) and *B*. *subtilis* (24 h) in chronic test. Unlike SA-SA mixtures, strong synergism was observed with SA-SAP, with TU ranging from 0.37–0.55. The synergistic effects between SAs and SAPs were due to their double blocking effects on the folate metabolism pathways (Figure S3). In detail, SAs inhibited the activity of DHPS, blocking the generation of dihydrofolate; while SAPs interfered with the activity of DHFR and blocked the biosynthesis of tetrahydrofolic acid. As a result, the SA-SAP mixtures lead to increased inhibition on the bacteria. With respect to the mixtures of SA-TC and SAP-TC, antagonistic effects were generally observed, which was in agreement with the results of the acute test. The antagonist effects of SA-TC and SAP-TC in the chronic test were probably due to the mutual effect of the two components. As noted in the study of Long *et al*.^26^, TCs can inhibit the protein synthesis and decrease the amount of intracellular DHPS or DHFR accordingly, which led to the decrease of the acting sites of SAs or SAPs and thus weakened their toxicity.

#### Quantitative relation between K_a_ and K_c_ for antibiotic mixtures

The K_a_ and K_c_ values for the binary antibiotic mixtures were calculated and the comparisons between them were shown in Fig. 2B. Similarly to the single toxicity, K_c_ for the mixtures was significantly greater than K_a_. Notably, mixtures of SA-SAP that presented synergistic joint effects showed the largest differences between K_a_ and K_c_, with K_a_/K_c_ ranging from 500 to 1500. For the mixtures of SA-TC that are antagonistic, the differences between K_a_ and K_c_ were relatively lower, which varied between 100 to 800 fold; while for SA-SA mixtures that showed additive effects, the differences between K_a_ and K_c_ were about 300 times.

Similarly, the docking-based descriptors were also employed to construct the QSTR model for ${\rm{lg}}({{\rm{K}}}_{{\rm{c}}}^{{\rm{m}}}/{{\rm{K}}}_{{\rm{a}}}^{{\rm{m}}})$, i.e., the differences between K_a_ and K_c_ for the mixtures (${{\rm{K}}}_{{\rm{c}}}^{{\rm{m}}}\,$and ${{\rm{K}}}_{{\rm{a}}}^{{\rm{m}}}$). The total mixture data (81) were split into training (80%, 64 data) and test (20%, 17 data) subsets randomly (see Tables S2 and S3, respectively). A model for ${\rm{lg}}({{\rm{K}}}_{{\rm{c}}}^{{\rm{m}}}/{{\rm{K}}}_{{\rm{a}}}^{{\rm{m}}})$ was developed based on training set using Ebind as the descriptors and the linear equation was as follows:

5 $$\begin{array}{rcl}{\rm{lg}}\,\frac{{{\rm{K}}}_{{\rm{c}}}^{{\rm{m}}}}{{{\rm{K}}}_{{\rm{a}}}^{{\rm{m}}}} & = & 1.009-0.007\frac{{{\rm{C}}}_{{\rm{A}}}^{{\rm{a}}}}{\sum {{\rm{C}}}^{{\rm{a}}}}{{\rm{E}}}_{{\rm{A}}}^{{\rm{Luc}}}-2.056\times {10}^{-4}\frac{{{\rm{C}}}_{{\rm{B}}}^{{\rm{a}}}}{\sum {{\rm{C}}}^{{\rm{a}}}}{{\rm{E}}}_{{\rm{B}}}^{{\rm{Luc}}}+0.021\frac{{{\rm{C}}}_{{\rm{A}}}^{{\rm{c}}}}{\sum {{\rm{C}}}^{{\rm{c}}}}{{\rm{E}}}_{{\rm{A}}}^{{\rm{LitR}}}\\  &  & +\,0.012\frac{{{\rm{C}}}_{{\rm{B}}}^{{\rm{c}}}}{\sum {{\rm{C}}}^{{\rm{c}}}}{{\rm{E}}}_{{\rm{B}}}^{{\rm{LitR}}}\end{array}$$

n = 64, R^2^ = 0.831, RMSE = 0.031, F = 72.657, P = 0.000, ${{\rm{Q}}}_{{\rm{loo}}}^{2}$ = 0.793, RMSE_loo_ = 0.033, ${{\rm{Q}}}_{{\rm{lmo}}}^{2}$ = 0.792, RMSE_lmo_ = 0.033, ${{\rm{Q}}}_{{\rm{F}}1}^{2}$ = 0.789, RMSEP = 0.016.

**Figure 5:**
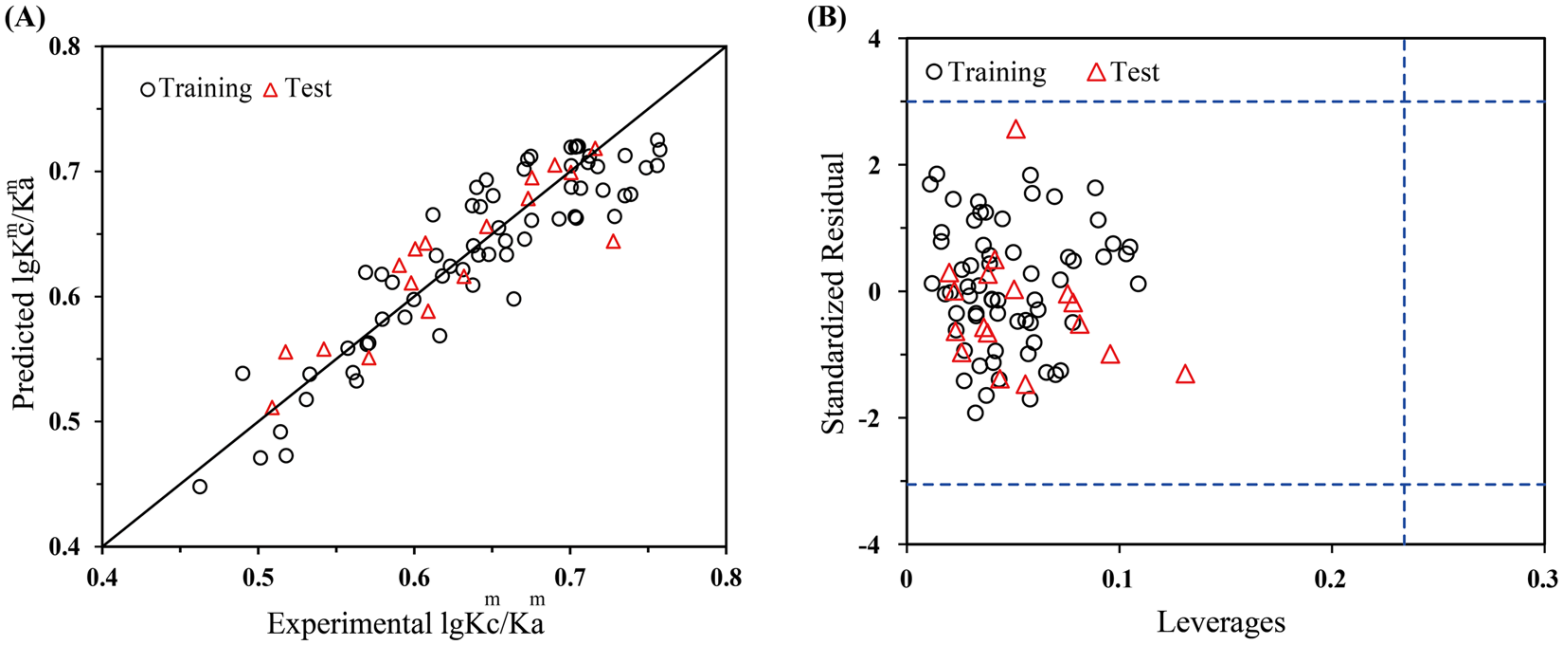
(**A**) Plots of experimental versus predicted ${\rm{lg}}({{\rm{K}}}_{{\rm{c}}}^{{\rm{m}}}/{{\rm{K}}}_{{\rm{a}}}^{{\rm{m}}})$ values. (**B)** Williams plot of the training and test sets with a warning leverage h* = 0.237. h* was calculated by h* = 3(m + 1)/n, where m is the number of the descriptors, and n is the number of the data.

As seen from Equation 5, the ${\rm{lg}}({{\rm{K}}}_{{\rm{c}}}^{{\rm{m}}}/{{\rm{K}}}_{{\rm{a}}}^{{\rm{m}}})$ values were correlated with four docking-based descriptors, *i*.*e*., the E^Luc^ and E^LitR^ of each component (A and B). The four descriptors represent the actions of component A and B in acute and chronic test, respectively. In this equation, ${{\rm{C}}}_{{\rm{A}}}^{{\rm{a}}}$ and ${{\rm{C}}}_{{\rm{B}}}^{{\rm{a}}}$ represented the acute EC_50_s of component A and B, while ${{\rm{C}}}_{{\rm{A}}}^{{\rm{c}}}$ and ${{\rm{C}}}_{{\rm{B}}}^{{\rm{c}}}$ denoted the chronic EC_50_s. The parameter ${C}_{i}/\sum C$ indicated the apparent concentration proportion of component *i* in the mixtures, which was proposed by Zou *et al*.^19^ and have been introduced for the QSTR model constructions for mixtures^26, 31^. This parameter was involved in this model to reflect the varying contribution of drug *i* in different mixtures. R^2^ = 0.831 of this model indicated high goodness-of-fit of the training set. Internal validation was carried out using leave-one-out (LOO) and leave-many-out (LMO) method. For the latter case, a group of data including 20% of the training set were left out and predicted later by the model obtained with the remaining 80% of the data. The higher ${{\rm{Q}}}_{{\rm{loo}}}^{2}$ (0.793) and ${{\rm{Q}}}_{{\rm{lmo}}}^{2}$ (0.792) suggested good robustness and stability of this model. The external predictive performance of this model was assessed by ${{\rm{Q}}}_{{\rm{F}}1}^{2}$ ^32^. The high value of ${{\rm{Q}}}_{{\rm{F}}1}^{2}$ suggested that the constructed model have good predictive performance. Plots of the experimental versus predicted ${\rm{lg}}({{\rm{K}}}_{{\rm{c}}}^{{\rm{m}}}/{{\rm{K}}}_{{\rm{a}}}^{{\rm{m}}})$ were presented in Fig. 5A. It can be seen that the predicted ${\rm{lg}}({{\rm{K}}}_{{\rm{c}}}^{{\rm{m}}}/{{\rm{K}}}_{{\rm{a}}}^{{\rm{m}}})$ values were in good correlation with the experimental values, for both the training and test set. The applicability domain (AD) for this model was characterized by the leverage approach^33^. A Williams plot of the leverage values versus standardized residuals for every data was obtained, as depicted in Fig. 5B. The Williams plot allows a graphical detection of both the outliers and the influential chemicals in a model^34^. According to Fig. 5B, all of the leverage values were less than the warning leverage (in this case h* = 0.237 as indicated by the vertical dash line), and there were no outlier data with standard residuals >3δ (indicated by the horizontal dash lines) for both the training and test sets. This suggested that all of the data fall inside the AD of the model, and the model can thus be utilized to predict the ${\rm{lg}}({{\rm{K}}}_{{\rm{c}}}^{{\rm{m}}}/{{\rm{K}}}_{{\rm{a}}}^{{\rm{m}}})$ values.

Other functional forms and descriptors were also investigated for the possibility to construct a QSTR model for the ${\rm{lg}}({{\rm{K}}}_{{\rm{c}}}^{{\rm{m}}}/{{\rm{K}}}_{{\rm{a}}}^{{\rm{m}}})$ values. For example, we used the moving average descriptors (ΔE_*ij*_) to construct a model as follows:

6 $${\rm{lg}}\,\frac{{{\rm{K}}}_{{\rm{c}}}^{{\rm{m}}}}{{{\rm{K}}}_{{\rm{a}}}^{{\rm{m}}}}=0.450-0.006{{\rm{\Delta }}{\rm{E}}}_{{\rm{A}}}^{{\rm{Luc}}}+0.005{{\rm{\Delta }}{\rm{E}}}_{{\rm{B}}}^{{\rm{Luc}}}+0.018{{\rm{\Delta }}{\rm{E}}}_{{\rm{A}}}^{{\rm{T}}}+0.005{{\rm{\Delta }}{\rm{E}}}_{{\rm{B}}}^{{\rm{T}}}$$

n = 64, R^2^ = 0.292, RMSE = 0.064, F = 6.093, P = 0.000

R^2^ = 0.292 indicated bad fitting of this model, therefore the moving average descriptors may not apply to the QSTR model construction for the mixture data.

### QSTR models for acute to chronic toxicity extrapolation

As depicted in Figure S4, the equation of the linear regression for the dose-response curves is denoted as:

7 $${\rm{Inhibition}}( \% )={\rm{K}}\times {\rm{lgC}}+{\rm{b}}$$

K is the slope of the fitting curve, and b denotes the intercept. Based on this equation, we can obtain the −lgEC_50_ for the acute and chronic toxicity:

8 $$-\,{{\rm{lgEC}}}_{50}^{{\rm{a}}}=\frac{50-{{\rm{b}}}_{{\rm{a}}}}{{{\rm{K}}}_{{\rm{a}}}},-{{\rm{lgEC}}}_{50}^{{\rm{c}}}=\frac{50-{{\rm{b}}}_{{\rm{c}}}}{{{\rm{K}}}_{{\rm{c}}}}$$

Then, the ratio of −${{\rm{lgEC}}}_{50}^{{\rm{a}}}$ to −${{\rm{lgEC}}}_{50}^{{\rm{c}}}$ can be calculated:

9 $$\frac{-{{\rm{lgEC}}}_{50}^{{\rm{a}}}}{-{{\rm{lgEC}}}_{50}^{{\rm{c}}}}=\frac{{{\rm{K}}}_{{\rm{c}}}}{{{\rm{K}}}_{{\rm{a}}}}\times \frac{50-{{\rm{b}}}_{{\rm{a}}}}{50-{{\rm{b}}}_{{\rm{c}}}}$$

Equation 9 can be transformed by taking the logarithm values of the two sides of the equation:

10 $$\mathrm{lg}(-{{\rm{lgEC}}}_{50}^{{\rm{a}}})-\,\mathrm{lg}(-{{\rm{lgEC}}}_{50}^{{\rm{c}}})=\,{\rm{lg}}\,\frac{{{\rm{K}}}_{{\rm{c}}}}{{{\rm{K}}}_{{\rm{a}}}}+\,{\rm{lg}}\,\frac{50-{{\rm{b}}}_{{\rm{a}}}}{50-{{\rm{b}}}_{{\rm{c}}}}$$

Particularly, we fitted ${\rm{lg}}\,\frac{{{\rm{K}}}_{{\rm{c}}}}{{{\rm{K}}}_{{\rm{a}}}}$ with ${\rm{lg}}\,\frac{50-{b}_{a}}{50-{b}_{c}}$ and found a good correlation between them:

11 $${\rm{lg}}\,\frac{50-{{\rm{b}}}_{{\rm{a}}}}{50-{{\rm{b}}}_{{\rm{c}}}}=1.059-2.504\,{\rm{lg}}\,\frac{{{\rm{K}}}_{{\rm{c}}}}{{{\rm{K}}}_{{\rm{a}}}}$$

n = 15, R^2^ = 0.703, RMSE = 0.198, F = 30.724, P = 0.000.

Taking Equations 1 and 11 into Equation 10, we finally obtained the prediction model for the acute to chronic toxicity extrapolation for individual antibiotics:

12 $$\mathrm{lg}(-\mathrm{lg}\,{{\rm{EC}}}_{50}^{{\rm{c}}})=\,\mathrm{lg}(-{{\rm{lgEC}}}_{50}^{{\rm{a}}})-0.006{{\rm{E}}}^{{\rm{Luc}}}-0.017{{\rm{E}}}^{{\rm{LitR}}}-0.645\,$$

For antibiotic mixtures, $\mathrm{lg}\,\frac{{K}_{{\rm{c}}}^{{\rm{m}}}}{{K}_{{\rm{a}}}^{{\rm{m}}}}$ and $\mathrm{lg}\,\frac{50-{b}_{{\rm{a}}}^{{\rm{m}}}}{50-{b}_{{\rm{c}}}^{{\rm{m}}}}$ also had a good correlation, which was as follows:

13 $${\rm{lg}}\,\frac{50-{b}_{a}^{m}}{50-{b}_{c}^{m}}=-0.093-1.101\,{\rm{lg}}\,\frac{{K}_{c}^{m}}{{K}_{a}^{m}}$$

n = 64, R^2^ = 0.971, RMSE = 0.075, F = 2099.31, P = 0.000, ${{\rm{Q}}}_{{\rm{loo}}}^{2}$ = 0.969, RMSE_loo_ = 0.076, ${{\rm{Q}}}_{{\rm{lmo}}}^{2}$ = 0.969, RMSE_lmo_ = 0.076, ${{\rm{Q}}}_{{\rm{F}}1}^{2}$ = 0.969, RMSEP = 0.040.

**Figure 6:**
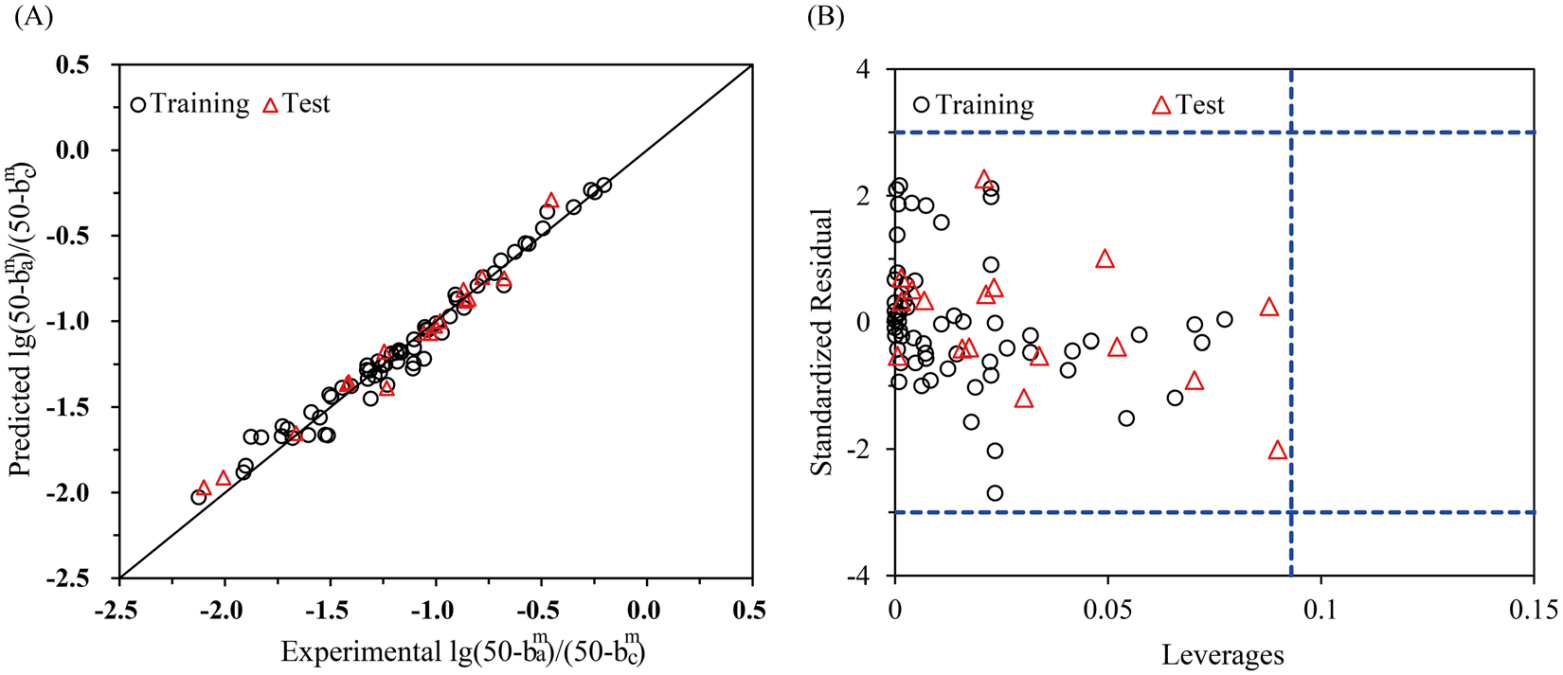
(**A**) Plots of experimental versus predicted ${\rm{lg}}\,\frac{50-{b}_{a}^{m}}{50-{b}_{c}^{m}}$ values. (**B**) Williams plot of the training and test sets with a warning leverage h* = 0.093.

The plots of the experimental versus predicted ${\rm{lg}}\,\frac{50-{b}_{a}^{m}}{50-{b}_{c}^{m}}$ values and the Williams plots of this model were depicted in Fig. 6A and B, respectively. The internal (${{\rm{Q}}}_{{\rm{loo}}}^{2}$ = 0.969 and ${{\rm{Q}}}_{{\rm{lmo}}}^{2}$ = 0.969) and external validation (${{\rm{Q}}}_{{\rm{F}}1}^{2}$ = 0.969) and the Williams plot of this model all confirm that this model could be utilized to predict the ${\rm{lg}}\,\frac{50-{b}_{a}^{m}}{50-{b}_{c}^{m}}$ values.

Likewise, taking Equations 5 and 13 into Equation 10, we obtained the final chronic toxicity extrapolation model for the mixtures:

14 $$\begin{array}{rcl}{\rm{lg}}(-{{\rm{lgEC}}}_{50}^{{\rm{m}},{\rm{c}}}) & = & \mathrm{lg}(-{{\rm{lgEC}}}_{50}^{{\rm{m}},{\rm{a}}})-0.0007\frac{{{\rm{C}}}_{{\rm{A}}}^{{\rm{a}}}}{\sum {{\rm{C}}}^{{\rm{a}}}}{{\rm{E}}}_{{\rm{A}}}^{{\rm{Luc}}}-2.077\times {10}^{-5}\frac{{{\rm{C}}}_{{\rm{B}}}^{{\rm{a}}}}{\sum {{\rm{C}}}^{{\rm{a}}}}{{\rm{E}}}_{{\rm{B}}}^{{\rm{Luc}}}\\  &  & +\,0.002\frac{{{\rm{C}}}_{{\rm{A}}}^{{\rm{c}}}}{\sum {{\rm{C}}}^{{\rm{c}}}}{{\rm{E}}}_{{\rm{A}}}^{{\rm{LitR}}}+0.001\frac{{{\rm{C}}}_{{\rm{B}}}^{{\rm{c}}}}{\sum {{\rm{C}}}^{{\rm{c}}}}{{\rm{E}}}_{{\rm{B}}}^{{\rm{LitR}}}+0.195\end{array}$$

**Figure 7:**
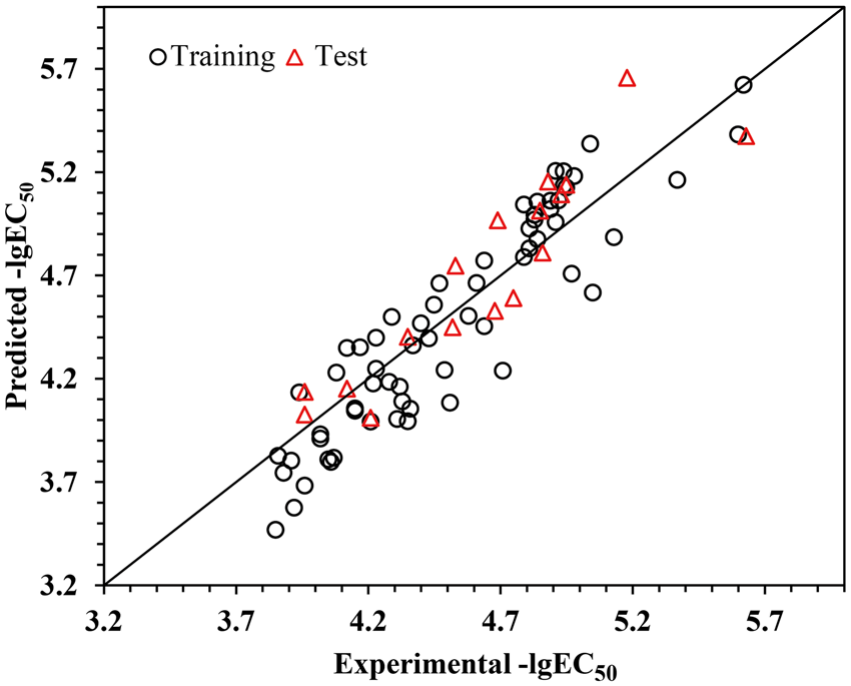
Plots of experimental versus predicted −${{\rm{lgEC}}}_{50}^{{\rm{m}},{\rm{c}}}$ by model 14.

This model was derived from model 5 and 13 that have been well established and validated. This model established a linkage between the chronic and acute mixture toxicity using the docking-based descriptors. Based on this model, we calculated the predicted −${{\rm{lgEC}}}_{50}^{{\rm{m}},{\rm{c}}}$ and plotted them versus the experimental −${{\rm{lgEC}}}_{50}^{{\rm{m}},{\rm{c}}}$ values, as shown in Fig. 7. It can be seen from Fig. 7 that there was satisfactory agreement between the observed and predicted values, which suggested that the model could be used to predict the mixture chronic toxicity of the antibiotics.

Moreover, in this model, $\mathrm{lg}(-{{\rm{lgEC}}}_{50}^{{\rm{c}}})$ is linearly related to $\,\mathrm{lg}(-{{\rm{lgEC}}}_{50}^{{\rm{a}}})$, which is different from the ACR model that describes a simple linear relationship between ${{\rm{EC}}}_{50}^{{\rm{c}}}$ and ${\,\text{EC}}_{50}^{{\rm{a}}}$. In addition, model 14 also differs from the model proposed by Zou *et al*.^19^, in which −${{\rm{lgEC}}}_{50}^{{\rm{c}}}$ was linearly correlated with −${{\rm{lgEC}}}_{50}^{{\rm{a}}}$. The complex relationship between ${{\rm{EC}}}_{50}^{{\rm{c}}}$ and ${\,\text{EC}}_{50}^{{\rm{a}}}$ as indicated by model 14 might be the reason for which the predecessors were not able to exactly extrapolate the chronic toxicity from corresponding acute data.

## Discussion

In this paper, we put forward a new model (Equation 12) for predicting the chronic toxicity of mixtures based on their acute toxicity. This model reflected a nonlinear correlation between the acute and chronic toxicity, which suggested that the lg(−lgEC_50_)s for the acute and chronic toxicity presented a good correlation. Compared to previous prediction models, this model was improved with regard to the following aspects.

### The present model was based on a good understanding on the toxic mechanisms

The previous models were usually based on a simple safety coefficient for the acute to chronic toxicity extrapolation, without considering the different toxicity mechanisms between the acute and the chronic toxicity. As a consequence, these methods can only apply to the narcotic compounds, whose toxicity effects are merely the baseline toxicity that is related to the lipid solubility as characterized by lgK_ow_. For example, Blaschke *et al*.^35^ found that the acute and chronic toxicity of narcotic compounds towards *Vibrio fischeri* both had a good correlation with lgK_ow_, with determination coefficients up to 0.95 and 0.94, respectively. In this sense, the ACRs for the narcotic compounds are usually constant, because their acute and chronic toxicity shared the same mechanism. Whereas, the ACRs for the reactive compounds (specific acting) may vary vastly (10–10000), because their acute and chronic toxic effects are induced by completely different mechanisms. For instance, Ahlers *et al*.^3^ discovered that the variations in ACRs of different reactive compounds can reach 5–6 orders of magnitude, even for the same species. Therefore, it is quite unreliable to simply extrapolate the chronic toxicity of reactive chemicals from the acute data by taking ACRs as a constant value.

In this study, we constructed the prediction model based on the premise that the targets of the chemicals in acute and chronic actions were identified, i.e., luciferase and LitR respectively. The toxic effects of the chemicals in acute and chronic actions were represented by their binding energies with the target proteins, which were used for building the prediction model. This mechanism-based extrapolation was proven to have good predictive capacity.

### The present model can apply to compounds with different action mechanisms

Zou *et al*.^19^ have developed a QSTR model for predicting the chronic mixture toxicity of SAs and TMP. In Zou’s model, the binding energies of SAs and TMP with luciferase were employed to represent their acute toxicity, while their binding energies with DHPS and DHFR were employed for their chronic toxicity, respectively. We attempted to verify Zou’s model for its ability to predict the chronic mixture toxicity of SAs and TCs, by replacing the binding energies of TMP (with DHFR) with the binding energies of TCs (with 30 s subunit), as indicated by Equation S1 in supporting information.

The comparison between the predicted and experimental values was shown in Figure S5, from which we can see that the predicted −lgEC_50_s were significantly greater than the experimental values. Therefore, Zou’s model is not suitable for predicting the chronic mixture toxicity of SAs and TCs, though it may have good predictive ability for SA-TMP mixtures. This limitation of Zou’s model is also the problem with many QSTR models, that is, a QSTR model can only apply to the compounds with the same action mechanism, while for compounds with different action mechanisms it may not work^20^.

In this research, the three types of antibiotics, i.e., SAs, SAPs and TCs, acted through different mechanisms during chronic test by targeting DHPS, DHFR and 30 s subunit, respectively. But in constructing the QSTR models for their binary mixture toxicity, we used their binding energies with LitR to substitute for the binding energies with their respective target proteins. This is feasible because LitR is likely involved in regulating the bacterial growth and thereby the production of the proteins including DHPS, DHFR and 30 s subunit. Particularly, we found a good correlation between the E_bind_ with LitR (E^LitR^) and the E_bind_ with the target proteins (E^T^) for the three types of antibiotics. As shown in Equation 15, the determination coefficient was up to 0.805.

15 $${E}^{{\rm{LitR}}}=1.725Ebin{d}^{{\rm{T}}}+20.107$$

n = 15, R^2^ = 0.805, RMSE = 0.016, F = 46.796, P = 0.000.

Therefore, the E^LitR^ can be used to substitute for the E^T^ in constructing the QSTR models. By doing so, we obtained the prediction model that can apply to the compounds with distinct mechanisms.

In particular, our model can be applicable for predicting the chronic toxicity of chemicals with unknown action mechanism, which is also another distinctive advantage of this model. Because this model utilizes E_bind_ with LitR as the characterization of chronic toxicity without considering the particular targets of the compounds, which makes the prediction model stand a good chance of applying to chemicals with unknown action mechanism.

### Limitations of the present model

All prediction models may have some intrinsic limitations from various aspects, and the limitations of this approach can be concluded as follows: firstly, this model can merely apply to binary mixtures at equitoxic ratio. The case of multicomponent mixtures with non-equitoxic ratio should be considered in the following research. What’s more, our model needs to be validated by a large amount of experimental data before it can be put into application confidently.

## Materials and Methods

### Chemicals and organisms

All of the chemicals were purchased from Aladdin Sigma-Aldrich (St. Louis, MO, USA) (purity ≥99%), as listed in Table 1. *V*. *fischeri* (AS 1.3842) was purchased from Species Conservation Center of Chinese Academy of Sciences.

### Toxicity test

The acute and chronic toxicity of the chemicals were determined based on a bioluminescence inhibition test, the detailed procedure was as follows: first, the chemicals were prepared into a series of solutions (in 2% NaCl) and added into the diluted bacteria suspension that has been cultured to exponential growth phase. DMSO was added to the chemical solutions with a final concentration of 0.5% v/v in order to enhance the dissolution of the chemicals. The 0.5% v/v DMSO induced no adverse effects on the bacterial growth and the bioluminescence^36^. For the chronic test, sufficient culture medium was added to support the growth of the bacteria; while in the acute test, the culture medium was replaced with 2% NaCl. After completely mixed, the bacteria suspensions were incubated at 20 °C for 15 min (acute) and 24 h (chronic) respectively. Afterwards, the inhibition rate of chemicals to the bacteria was calculated by the following equation:

16 $${\rm{Inhibition}} \% =\frac{{{\rm{L}}}_{0}-{{\rm{L}}}_{{\rm{i}}}}{{{\rm{L}}}_{0}}\times 100 \% $$

where *L*_*0*_ represents the light intensity of the control group, and *L*_*i*_ denotes the light intensity of the exposed group *i*. Then the dose-response curve was plotted with the compounds’ concentration and their inhibition on the bioluminescence, and the EC_50_ for acute toxicity of each compound was calculated.

For the mixture toxicity test, the binary mixtures were prepared at equitoxic ratio according to the individual EC_50_s of the components. Then the mixture toxicity was determined based on the above method and the EC_50_ for the mixtures (EC_50m_) were obtained. The joint effects of the antibiotic mixtures are characterized by the total toxicity unit (TU) as calculated by ${\rm{TU}}=\sum \frac{{{\rm{C}}}_{{\rm{i}}}}{{{\rm{EC}}}_{50,{\rm{i}}}}$, where C_*i*_ was the concentration of each component when the inhibition rate of the mixture achieved 50%; EC_50,*i*_ represented EC_50_ of the individual component *i*. TU ranging from 0.8 to 1.2 represents additive joint effect, while TU > 1.2 indicates antagonism and TU < 0.8 synergism^37^.

### Homology Modeling

The crystal structures of the proteins are required for the protein-chemical docking studies. For luciferase, LitR and the 30 s subunit of ribosomes, their crystal structures (3FGC, 3WHP and 4U1U, respectively) were directly obtained from the protein data bank (PDB, http://www.pdb.org). While the crystal structures of dihydropteroate synthase (DHPS) and dihydrofolate reductase (DHFR) were constructed using Homology Modeling module in Discovery Studio 3.1 (DS3.1, Accelrys Software Inc., San Diego, CA). The protein sequences YP_203863.1 (DHPS) and Q5E7M1 (DHFR) were obtained from NCBI (https://www.ncbi.nlm.nih.gov), which were selected as the target sequences for the homology modeling. BLAST (Basic Local Alignment Search Tool) at the NCBI website (http://blast.ncbi.nlm.nih.gov/Blast.cgi) was then run with the two sequences over the protein database bank, based on which 1AJ0 and 3TYU were selected as the templates for DHPS and DHFR, respectively. Afterwards, Align Sequence to the Templates wizard was performed on DS3.1, followed by the Building Homology Model. The “copy ligand” function was employed during the modeling, which embedded the ligand of the templates into the modeled proteins. In the Building Homology Model protocol, the number of models was set as 20, which generates 20 modeled structures for each protein. These structures were then subjected to Verify Protein (MODELER), and the structure with the highest Verify Score was chosen for the docking studies after the Loop Refinement (MODDLER).

### Molecular Docking

Molecular Docking was performed by CDOCKER module in DS3.1. The crystal structures of the proteins are in complex with their ligands. Their active sites were defined by selecting the ligands as the centers with the radius of the site sphere at 11.0 Å. Prior to the docking work, the proteins were prepared by the protein preparation wizard, and the chemicals were subjected to the energy minimization. Then the CDOCKER docking was performed with the default parameters. The CDOCKER used the soft-core potentials with an optional grid representation to dock ligands into the active site of the receptor. 10 random conformers were generated for each compound, and the lowest CDOCKER interaction energy (Ebind) was selected to represent its binding affinity with the receptor.

### Data Analysis

Multiple linear regressions were performed using SPSS 18.0 (SPSS Inc.). The statistical quality of the fitted models was evaluated by the square of the correlation coefficient (R^2^), root mean standard error (RMSE), Fischer ratio (F), and the significant level (P). Cross-validation was employed for the internal validation of the constructed models using LOO (${{\rm{Q}}}_{{\rm{loo}}}^{2}$), LTO (${{\rm{Q}}}_{{\rm{lto}}}^{2}$) and LMO (${{\rm{Q}}}_{{\rm{lmo}}}^{2}$) methods. In the cross-validation, different proportions of data (one, two and many for LOO, LTO and LMO, respectively) are iteratively held-out from the training set and predicted as new by the developed model in order to verify internal “predictivity”^38^. Q^2^ was calculated by the following equation:

17 $${Q}^{2}=1-\frac{{\sum }^{}{({y}_{i}-\overline{{y}_{i}})}^{2}}{{\sum }^{}{({y}_{i}-{y}_{mean})}^{2}}$$

where y_*i*_, and $\overline{{y}_{i}}$ are the actual and predicted values of the dependent variables in the training set, respectively; y_*mean*_ is the average value of all the dependent variables in the training set. The external validation of the model was characterized by ${{\rm{Q}}}_{{\rm{F}}1}^{2}$ ^39^, which was calculated as follows:

18 $${Q}_{F1}^{2}=1-\frac{\sum {({Y}_{i}-\overline{{Y}_{i}})}^{2}}{\sum {({Y}_{i}-{y}_{mean})}^{2}}$$

where *Y*_*i*_ and $\overline{{Y}_{i}}$ are the actual and predicted values of the dependent variables in the test set, respectively; y_*mean*_ is the average value of all the dependent variables in the training set.

## Conclusion

In the current work, a QSTR model was built for predicting the chronic mixture toxicity of antibiotics on bioluminescence based on the acute data and the docking-based descriptors. This model revealed a complex relationship between the acute and chronic toxicity, i.e. the lg(−lgEC_50_)s for the acute and chronic toxicity were linearly correlated. This is different from the ACR prediction method that describes a simple linear relationship between EC_50_s for the acute and chronic toxicity. The present model was based on a good understanding on the differences between the acute and chronic action mechanisms. In particular, the interaction energies (E_bind_) of the chemicals with LitR, rather than their respective target proteins, were introduced to represent their toxic effects in the chronic test. Therefore, the present model could probably apply to chemicals with distinct toxic mechanisms as well as those with undefined toxic mechanism. This breaks the limitation of the traditional QSTR models that can only apply to chemicals with similar structures or the same toxic mechanisms. Although the prediction capacity of the present model still needs further validation, it may provide a novel idea for the acute to chronic toxicity extrapolation studies, which may help with the environmental risk assessment on the pollutants.

## Electronic supplementary material

supplementary information
