# Supplementary material for: A Mechanism-based QSTR Model for Acute to Chronic Toxicity Extrapolation: A Case Study of Antibiotics on Luminous Bacteria
Source: Sci Rep. 2017 Jul 20;7:6022. doi: 10.1038/s41598-017-06384-9 (PMC5519556; doi:10.1038/s41598-017-06384-9)
Supplement: Supplementary file 1 — supplementary information [file 41598_2017_6384_MOESM1_ESM.doc]

**A Mechanism-based QSTR Model for Acute to Chronic Toxicity Extrapolation: A Case Study of Antibiotics on Luminous Bacteria**

Dali Wang^a,b^, Yue Gu^c^, Min Zheng^a^, Wei Zhang^d^, Zhifen Lin^a,e,f*^, Ying Liu^f^

# Supplementary Information

Enclosed:

**Figure S1.** The bioluminescence (line A) and OD (line B) curves of *V. fischeri* during 0-34 h

**Figure S2.** QS of V. fischeri

**Figure S3.** Schematic diagram for the mechanisms of the acute and chronic mixture toxicity

**Figure S4.** Linear fitting of the acute and chronic dose-response curves

**Equation S1.**

**Figure S5.** Plot of predicted –lgEC_50_ by Equation S1

**Table S1.** Acute and chronic toxicity of binary antibiotic mixtures

**Table S2.** The training set of the mixture toxicity data

**Table S3.** The test set of the mixture toxicity data


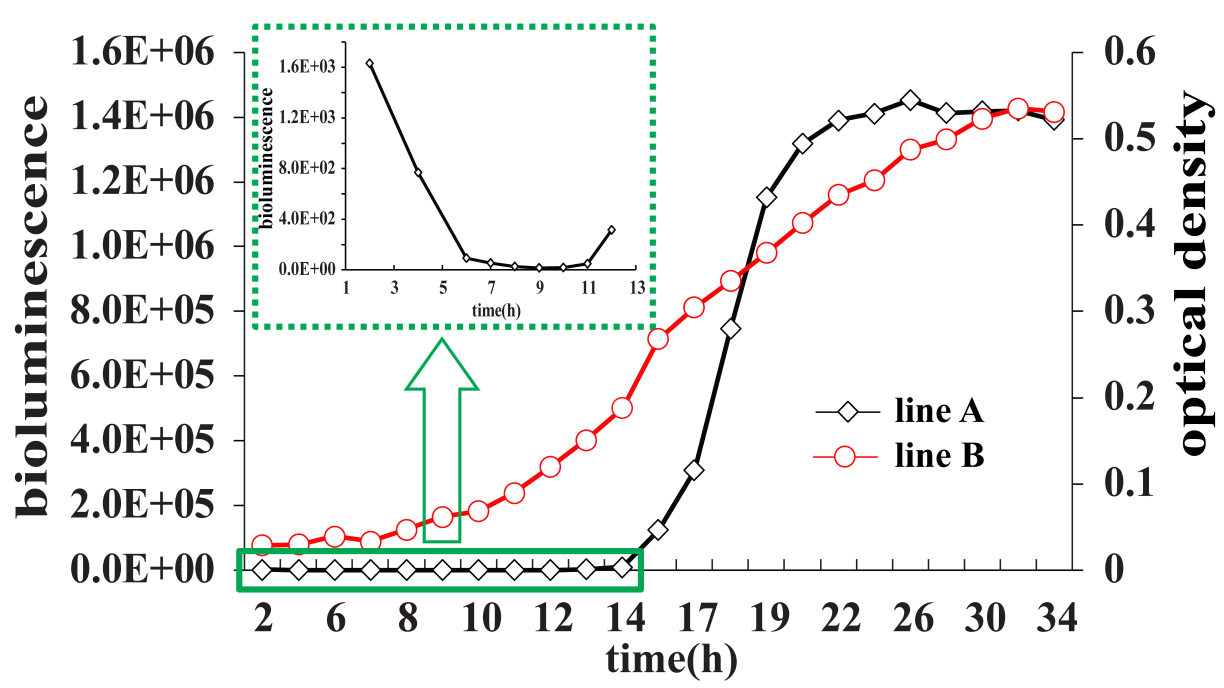


Figure S1. The bioluminescence (line A) and OD (line B) curves of *V. fischeri* during 0-34 h


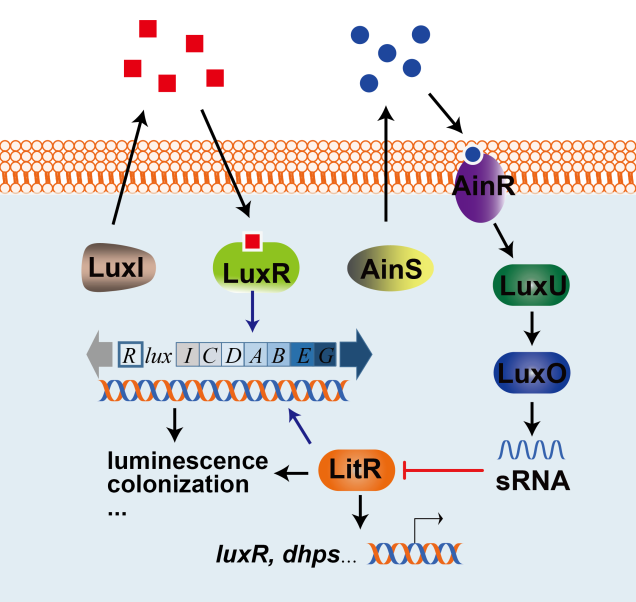


Figure S2. QS of *V. fischeri*. The bioluminescence of *V. fischeri* is regulated by two QS signal paths, namely the *luxI/R* and *ainS/R* systems ^1^. The two QS systems are mediated by the signal molecules C6-HSL and C8-HSL that are produced by LuxI and AinS respectively. These signal molecules accumulate around the bacteria and reenter into the intracellular once reach a certain threshold. The signal molecules then bind with LuxR and AinR respectively, switching on the expression of related genes and regulating the bioluminescence ultimately (Fig. 3). It was noteworthy that *luxI/R* and *ainS/R* systems are connected by LitR that can regulate the *lux* genes. Particularly, LitR may also play a role in controlling the growth of bacteria which needs folate metabolism, thus it is reasonable to speculate that LitR could participate in regulating the mRNA expression of *dhps* as well as other genes.


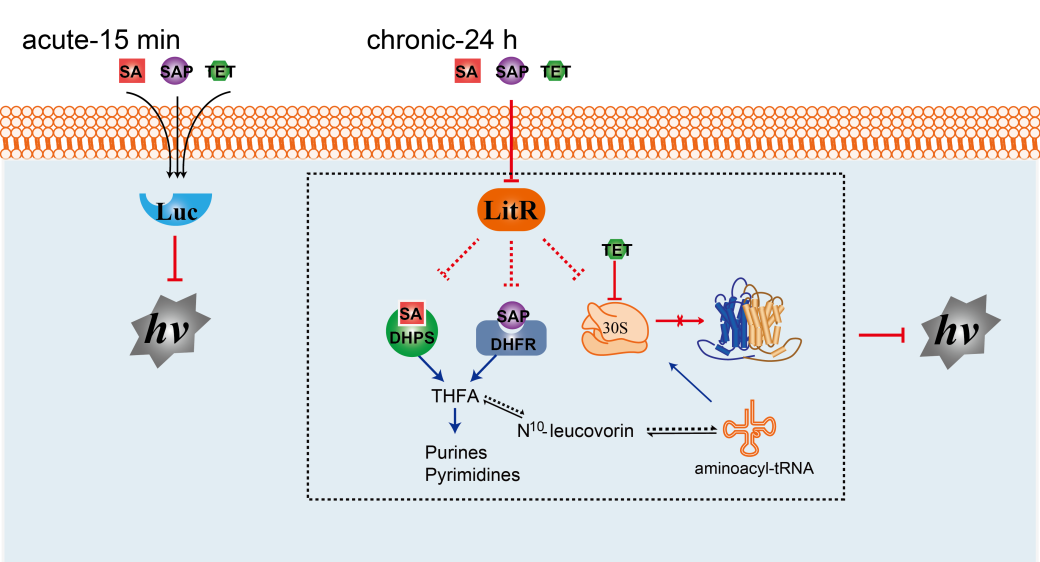


Figure S3. Schematic diagram for the mechanisms of the acute and chronic mixture toxicity


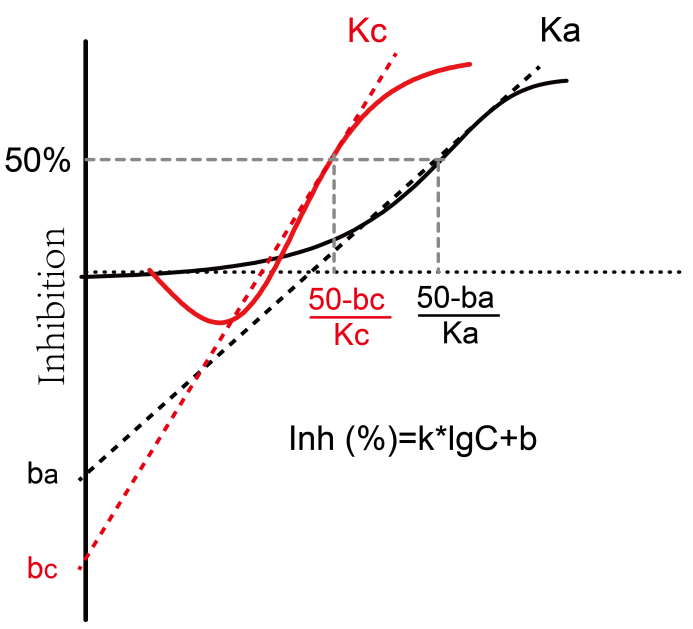


Figure S4. Linear fitting of the acute and chronic dose-response curves

Equation S1:

$\begin{matrix} -lgEC_{50,c}^{m}=-lgEC_{50,a}^{m}-0.027\frac{C_{SA}^{a}}{\sum C^{a}}Ebind_{SA}^{DHPS}-0.056\frac{C_{TET}^{a}}{\sum C^{a}}Ebind_{TC}^{30s} \\ -0.027\frac{C_{SA}^{c}}{\sum C^{c}}Ebind_{SA}^{Luc}+0.001\frac{C_{TC}^{c}}{\sum C^{c}}Ebind_{TCs}^{Luc}-0.220pKa+1.715 \end{matrix}$ (S1)


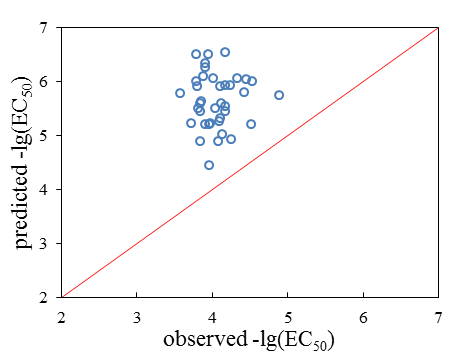


Figure S5. Plot of predicted –lgEC_50_ by Equation 14

Table S1. Acute and chronic TU of binary antibiotic mixtures

| No. | A | B | Acute | | Chronic | |  |
| --- | --- | --- | --- | --- | --- | --- | --- |
|  |  |  | TU | Effect | TU | Effect | |
| 1 | SD | SDX | 2.38 | antagonism | 1.03 | addition | |
| 2 |  | SM | 1.70 | antagonism | 0.81 | addition | |
| 3 |  | SMM | 1.68 | antagonism | 1.00 | addition | |
| 4 |  | SMP | 1.55 | antagonism | 1.08 | addition | |
| 5 |  | SMX | 1.42 | antagonism | 0.83 | addition | |
| 6 |  | SMZ | 1.88 | antagonism | 1.02 | addition | |
| 7 |  | SIX | 2.45 | antagonism | 0.99 | addition | |
| 8 | SDX | SM | 1.43 | antagonism | 0.98 | addition | |
| 9 |  | SMM | 1.68 | antagonism | 1.00 | addition | |
| 10 |  | SMP | 1.35 | antagonism | 1.09 | addition | |
| 11 |  | SMZ | 2.87 | antagonism | 0.96 | addition | |
| 12 | SMM | SMP | 1.33 | antagonism | 1.18 | addition | |
| 13 |  | SMX | 3.13 | antagonism | 1.08 | addition | |
| 14 |  | SMZ | 1.38 | antagonism | 1.15 | addition | |
| 15 |  | SM | 1.84 | antagonism | 1.02 | addition | |
| 16 | SMP | SMX | 1.27 | antagonism | 0.96 | addition | |
| 17 |  | SMZ | 1.29 | antagonism | 1.12 | addition | |
| 18 |  | SIX | 2.08 | antagonism | 1.09 | addition | |
| 19 |  | SM | 1.34 | antagonism | 0.87 | addition | |
| 20 | SMX | SMZ | 2.17 | antagonism | 1.04 | addition | |
| 21 |  | SIX | 1.46 | antagonism | 1.19 | addition | |
| 22 |  | SM | 1.59 | antagonism | 0.99 | addition | |
| 23 | SMZ | SIX | 1.41 | antagonism | 0.84 | addition | |
| 24 |  | SM | 1.64 | antagonism | 1.09 | addition | |
| 25 | SIX | SM | 1.29 | antagonism | 1.11 | addition | |
| 26 | SD | OMP | 4.00 | antagonism | 0.49 | synergism | |
| 27 |  | TMP | 2.98 | antagonism | 0.55 | synergism | |
| 28 | SDX | TMP | 3.38 | antagonism | 0.48 | synergism | |
| 29 |  | OMP | 3.58 | antagonism | 0.37 | synergism | |
| 30 | SM | TMP | 1.30 | antagonism | 0.48 | synergism | |
| 31 |  | OMP | 2.11 | antagonism | 0.39 | synergism | |
| 32 | SMM | TMP | 2.30 | antagonism | 0.42 | synergism | |
| 33 |  | OMP | 2.31 | antagonism | 0.32 | synergism | |
| 34 | SMP | TMP | 1.91 | antagonism | 0.52 | synergism | |
| 35 |  | OMP | 1.94 | antagonism | 0.35 | synergism | |
| 36 | SMX | TMP | 3.23 | antagonism | 0.54 | synergism | |
| 37 |  | OMP | 2.84 | antagonism | 0.46 | synergism | |
| 38 | SMZ | TMP | 1.23 | antagonism | 0.53 | synergism | |
| 39 |  | OMP | 4.65 | antagonism | 0.41 | synergism | |
| 40 | SIX | TMP | 2.06 | antagonism | 0.55 | synergism | |
| 41 |  | OMP | 2.92 | antagonism | 0.41 | synergism | |
| 42 | SD | TH | 2.3 | antagonism | 2.07 | antagonism | |
| 43 |  | CH | 1.83 | antagonism | 1.87 | antagonism | |
| 44 |  | OH | 1.76 | antagonism | 2.38 | antagonism | |
| 45 |  | DH | 1.42 | antagonism | 2.55 | antagonism | |
| 46 |  | MH | 2.47 | antagonism | 2.12 | antagonism | |
| 47 | SDX | TH | 2.29 | antagonism | 1.64 | antagonism | |
| 48 |  | CH | 1.65 | antagonism | 1.53 | antagonism | |
| 49 |  | OH | 2.77 | antagonism | 1.98 | antagonism | |
| 50 |  | DH | 2.19 | antagonism | 1.82 | antagonism | |
| 51 |  | MH | 1.95 | antagonism | 1.84 | antagonism | |
| 52 | SM | TH | 1.88 | antagonism | 1.42 | antagonism | |
| 53 |  | CH | 1.73 | antagonism | 2.21 | antagonism | |
| 54 |  | OH | 2.73 | antagonism | 1.37 | antagonism | |
| 55 |  | DH | 1.84 | antagonism | 1.91 | antagonism | |
| 56 |  | MH | 1.38 | antagonism | 1.56 | antagonism | |
| 57 | SMM | TH | 1.29 | antagonism | 1.40 | antagonism | |
| 58 |  | CH | 1.64 | antagonism | 1.42 | antagonism | |
| 59 |  | OH | 1.26 | antagonism | 1.72 | antagonism | |
| 60 |  | DH | 2.78 | antagonism | 2.11 | antagonism | |
| 61 |  | MH | 1.32 | antagonism | 2.55 | antagonism | |
| 62 | SMP | TH | 2.31 | antagonism | 1.45 | antagonism | |
| 63 |  | CH | 1.75 | antagonism | 1.29 | antagonism | |
| 64 |  | OH | 3.2 | antagonism | 1.85 | antagonism | |
| 65 |  | DH | 1.80 | antagonism | 1.51 | antagonism | |
| 66 |  | MH | 2.03 | antagonism | 2.41 | antagonism | |
| 67 | SMX | TH | 1.31 | antagonism | 1.27 | antagonism | |
| 68 |  | CH | 1.25 | antagonism | 1.41 | antagonism | |
| 69 |  | OH | 1.36 | antagonism | 1.95 | antagonism | |
| 70 |  | DH | 1.25 | antagonism | 1.58 | antagonism | |
| 71 |  | MH | 1.74 | antagonism | 1.91 | antagonism | |
| 72 | SMZ | TH | 2.03 | antagonism | 1.35 | antagonism | |
| 73 |  | CH | 2.01 | antagonism | 1.67 | antagonism | |
| 74 |  | OH | 1.98 | antagonism | 1.33 | antagonism | |
| 75 |  | DH | 1.46 | antagonism | 2.25 | antagonism | |
| 76 |  | MH | 1.51 | antagonism | 1.50 | antagonism | |
| 77 | SIX | TH | 1.39 | antagonism | 1.54 | antagonism | |
| 78 |  | CH | 1.51 | antagonism | 1.61 | antagonism | |
| 79 |  | OH | 1.65 | antagonism | 1.46 | antagonism | |
| 80 |  | DH | 1.61 | antagonism | 2.47 | antagonism | |
| 81 |  | MH | 2.01 | antagonism | 1.78 | antagonism | |

Table S2 Training set of the mixture toxicity data

| Mixtures | 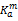 | 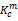 | 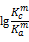 | 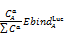 | 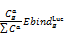 | 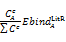 | 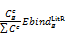 |
| --- | --- | --- | --- | --- | --- | --- | --- |
| SD+SIX | 42.74 | 232.35 | 0.74 | -24.30 | -8.06 | -17.73 | -7.99 |
| SD+SMM | 52.54 | 246.26 | 0.67 | -18.61 | -14.25 | -20.63 | -5.27 |
| SD+SMP | 51.30 | 219.46 | 0.63 | -15.19 | -18.18 | -20.07 | -6.34 |
| SD+SMX | 43.44 | 238.12 | 0.74 | -25.06 | -5.86 | -21.98 | -3.53 |
| SD+SMZ | 35.41 | 161.62 | 0.66 | -11.27 | -21.86 | -13.76 | -14.18 |
| SDX+SM | 59.76 | 202.95 | 0.53 | -8.26 | -29.70 | -20.06 | -11.18 |
| SDX+SMP | 46.24 | 146.74 | 0.50 | -10.13 | -24.37 | -26.46 | -4.88 |
| SIX+SMZ | 60.88 | 231.06 | 0.58 | -4.95 | -28.95 | -8.92 | -20.39 |
| SM+SMM | 58.41 | 245.32 | 0.62 | -26.40 | -11.29 | -23.62 | -6.32 |
| SM+SMP | 50.08 | 231.01 | 0.66 | -22.40 | -14.98 | -22.87 | -7.56 |
| SM+SMZ | 59.33 | 259.73 | 0.64 | -17.40 | -18.87 | -14.77 | -15.95 |
| SMM+SMP | 49.51 | 225.59 | 0.66 | -13.51 | -21.12 | -12.94 | -15.99 |
| SMM+SMX | 54.33 | 280.24 | 0.71 | -24.90 | -7.60 | -16.56 | -10.43 |
| SMM+SMZ | 58.94 | 241.27 | 0.61 | -9.62 | -24.37 | -5.99 | -24.19 |
| SD+TMP | 72.12 | 277.99 | 0.59 | -18.97 | -15.45 | -25.32 | -0.18 |
| SDX+OMP | 77.81 | 256.49 | 0.52 | -17.49 | -18.27 | -31.48 | -0.11 |
| SDX+TMP | 88.40 | 256.49 | 0.46 | -13.82 | -22.62 | -31.47 | -0.13 |
| SIX+OMP | 77.75 | 289.57 | 0.57 | -14.16 | -22.19 | -26.00 | -0.34 |
| SIX+TMP | 63.23 | 230.01 | 0.56 | -10.71 | -26.29 | -25.98 | -0.40 |
| SM+OMP | 65.73 | 249.69 | 0.58 | -30.21 | -8.78 | -30.39 | -0.19 |
| SMM+OMP | 61.30 | 240.75 | 0.59 | -21.27 | -14.44 | -27.14 | -0.63 |
| SMP+OMP | 64.40 | 239.16 | 0.57 | -24.88 | -10.81 | -29.39 | -0.56 |
| SMP+TMP | 66.96 | 206.95 | 0.49 | -21.51 | -14.63 | -29.35 | -0.65 |
| SMX+TMP | 79.03 | 288.96 | 0.56 | -7.86 | -27.39 | -24.96 | -1.09 |
| SMZ+TMP | 73.17 | 249.69 | 0.53 | -24.69 | -10.37 | -30.65 | -0.21 |
| SD+CH | 63.34 | 322.45 | 0.71 | -29.36 | -4.55 | -21.24 | -6.91 |
| SD+DH | 147.23 | 739.12 | 0.70 | -30.63 | -1.89 | -20.07 | -8.15 |
| SD+MH | 102.45 | 527.12 | 0.71 | -30.11 | -3.23 | -15.48 | -15.70 |
| SD+OH | 98.53 | 436.36 | 0.65 | -26.80 | -7.92 | -8.62 | -26.99 |
| SD+TH | 105.20 | 589.84 | 0.75 | -28.45 | -5.47 | -13.76 | -18.12 |
| SDX+CH | 63.34 | 261.84 | 0.62 | -28.68 | -8.93 | -27.62 | -5.24 |
| SDX+DH | 185.35 | 737.57 | 0.60 | -31.23 | -3.88 | -26.46 | -6.27 |
| SDX+OH | 98.53 | 365.22 | 0.57 | -24.15 | -14.34 | -13.09 | -23.91 |
| SDX+TH | 61.94 | 268.96 | 0.64 | -26.98 | -10.43 | -19.55 | -15.03 |
| SIX+CH | 50.31 | 269.25 | 0.73 | -26.93 | -12.57 | -18.08 | -13.04 |
| SIX+MH | 89.23 | 465.72 | 0.72 | -28.91 | -9.34 | -10.61 | -23.88 |
| SIX+OH | 49.38 | 233.85 | 0.68 | -21.31 | -18.97 | -4.81 | -33.36 |
| SIX+TH | 41.88 | 220.39 | 0.72 | -24.73 | -14.33 | -8.92 | -26.06 |
| SM+DH | 165.19 | 738.88 | 0.65 | -38.31 | -1.32 | -22.87 | -9.72 |
| SM+MH | 89.23 | 508.60 | 0.76 | -37.85 | -2.27 | -16.88 | -17.94 |
| SM+OH | 51.71 | 261.85 | 0.70 | -34.83 | -5.75 | -8.85 | -29.01 |
| SM+TH | 67.92 | 319.71 | 0.67 | -36.36 | -3.91 | -14.77 | -20.39 |
| SMM+CH | 50.31 | 273.38 | 0.74 | -30.75 | -6.22 | -15.02 | -19.13 |
| SMM+DH | 147.23 | 738.89 | 0.70 | -32.61 | -2.63 | -12.94 | -20.57 |
| SMM+MH | 89.23 | 508.85 | 0.76 | -31.83 | -4.46 | -7.39 | -29.37 |
| SMM+OH | 85.82 | 401.87 | 0.67 | -27.20 | -10.50 | -2.97 | -36.45 |
| SMM+TH | 61.95 | 314.44 | 0.71 | -29.46 | -7.40 | -5.99 | -30.91 |
| SMP+MH | 89.23 | 510.58 | 0.76 | -33.11 | -2.96 | -8.80 | -28.27 |
| SMP+OH | 47.16 | 223.07 | 0.67 | -29.74 | -7.34 | -3.62 | -35.89 |
| SMP+TH | 59.16 | 298.96 | 0.70 | -31.42 | -5.05 | -7.18 | -29.98 |
| SMX+CH | 74.41 | 323.36 | 0.64 | -21.10 | -13.99 | -11.47 | -23.19 |
| SMX+DH | 147.23 | 739.14 | 0.70 | -24.20 | -6.40 | -9.60 | -24.22 |
| SMX+OH | 98.53 | 438.05 | 0.65 | -16.31 | -20.62 | -1.94 | -37.76 |
| SMX+TH | 160.07 | 809.88 | 0.70 | -19.20 | -15.80 | -4.06 | -33.24 |
| SMZ+CH | 145.10 | 602.28 | 0.62 | -32.37 | -2.59 | -25.01 | -7.89 |
| SMZ+OH | 98.53 | 430.16 | 0.64 | -30.71 | -4.68 | -9.36 | -28.43 |
| SMZ+TH | 191.44 | 840.64 | 0.64 | -31.79 | -3.15 | -15.43 | -19.72 |
| SDX+SMM | 77.82 | 254.29 | 0.51 | -13.44 | -20.68 | -27.01 | -4.03 |
| SM+SMX | 55.93 | 252.29 | 0.65 | -33.16 | -4.33 | -25.51 | -4.30 |
| SMP+SMZ | 54.30 | 235.58 | 0.64 | -13.05 | -21.16 | -7.18 | -23.45 |
| SD+OMP | 67.59 | 277.99 | 0.61 | -22.14 | -11.51 | -25.33 | -0.15 |
| SM+TMP | 69.16 | 249.69 | 0.56 | -26.80 | -12.20 | -30.38 | -0.22 |
| SMP+CH | 50.31 | 248.12 | 0.69 | -32.35 | -4.19 | -17.21 | -17.74 |
| SMZ+MH | 104.83 | 529.24 | 0.70 | -32.84 | -1.81 | -17.54 | -17.27 |

Table S3 Test set of the mixture toxicity data

| Mixtures | | 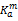 | 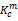 | | 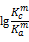 | | 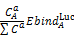 | | 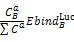 | | 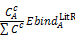 | | 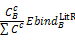 | |
| --- | --- | --- | --- | --- | --- | --- | --- | --- | --- | --- | --- | --- | --- | --- |
| SD+SDX | 49.43 | | | 257.08 | | 0.72 | | -21.66 | | -10.77 | | -10.70 | | -18.32 |
| SDX+SMZ | 33.16 | | | 107.06 | | 0.51 | | -6.91 | | -26.93 | | -19.55 | | -11.76 |
| SIX+SM | 60.24 | | | 234.66 | | 0.59 | | -6.01 | | -32.41 | | -9.33 | | -19.75 |
| SIX+SMM | 58.64 | | | 232.46 | | 0.60 | | -10.37 | | -23.93 | | -17.12 | | -9.69 |
| SIX+SMP | 47.23 | | | 191.97 | | 0.61 | | -7.53 | | -27.15 | | -16.29 | | -11.41 |
| SMP+SMX | 45.14 | | | 213.8 | | 0.68 | | -27.94 | | -5.45 | | -18.84 | | -9.60 |
| SMX+SMZ | 58.32 | | | 236.14 | | 0.61 | | -3.55 | | -29.50 | | -4.06 | | -26.01 |
| SMM+TMP | 69.1 | | | 240.75 | | 0.54 | | -17.58 | | -18.70 | | -27.10 | | -0.74 |
| SMX+OMP | 77.6 | | | 288.96 | | 0.57 | | -10.54 | | -23.44 | | -25.02 | | -0.93 |
| SMZ+OMP | 75.83 | | | 249.69 | | 0.52 | | -27.31 | | -7.32 | | -30.66 | | -0.18 |
| SDX+MH | 102.45 | | | 439.11 | | 0.63 | | -30.15 | | -6.49 | | -21.53 | | -12.75 |
| SM+CH | 63.33 | | | 280.62 | | 0.65 | | -37.18 | | -3.22 | | -24.47 | | -8.34 |
| SMP+DH | 147.23 | | | 738.93 | | 0.70 | | -33.64 | | -1.74 | | -14.98 | | -19.27 |
| SMX+MH | 112.33 | | | 529.19 | | 0.67 | | -22.84 | | -10.47 | | -5.10 | | -32.14 |
| SMZ+DH | 185.35 | | | 739.14 | | 0.60 | | -33.16 | | -1.06 | | -23.45 | | -9.24 |
| SD+SM | 47.5 | | | 253.72 | | 0.73 | | -13.00 | | -23.25 | | -14.19 | | -13.54 |
| SIX+DH | 150.66 | | | 738.24 | | 0.69 | | -30.43 | | -5.66 | | -16.29 | | -14.67 |

1. Lupp, C. & Ruby, E. G. Vibrio fischeri Uses Two Quorum-Sensing Systems for the Regulation of Early and Late Colonization Factors*. J. Bacteriol*. **18**7, 3620–3629 (2005).
